# Cellular Electrical Impedance as a Method to Decipher CCR7 Signalling and Biased Agonism

**DOI:** 10.3390/ijms23168903

**Published:** 2022-08-10

**Authors:** Nathan Vanalken, Katrijn Boon, Jordi Doijen, Dominique Schols, Tom Van Loy

**Affiliations:** 1 Laboratory of Virology and Chemotherapy, Department of Microbiology, Immunology and Transplantation, Rega Institute, KU Leuven, Herestraat 49, 3000 Leuven, Belgium; 2 Janssen Pharmaceutica NV, Turnhoutseweg 30, 2340 Beerse, Belgium

**Keywords:** G protein-coupled receptor, C-C chemokine receptor 7, cellular electrical impedance, label-free, G protein, β-arrestin, signalling, biased agonism

## Abstract

The human C-C chemokine receptor type 7 (CCR7) has two endogenous ligands, C-C chemokine ligand 19 (CCL19) and CCL21, displaying biased agonism reflected by a pronounced difference in the level of β-arrestin recruitment. Detecting this preferential activation generally requires the use of separate, pathway-specific label-based assays. In this study, we evaluated an alternative methodology to study CCR7 signalling. Cellular electrical impedance (CEI) is a label-free technology which yields a readout that reflects an integrated cellular response to ligand stimulation. CCR7-expressing HEK293 cells were stimulated with CCL19 or CCL21, which induced distinct impedance profiles with an apparent bias during the desensitisation phase of the response. This discrepancy was mainly modulated by differential β-arrestin recruitment, which shaped the impedance profile but did not seem to contribute to it directly. Pathway deconvolution revealed that Gαi-mediated signalling contributed most to the impedance profile, but Gαq- and Gα12/13-mediated pathways were also involved. To corroborate these results, label-based pathway-specific assays were performed. While CCL19 more potently induced β-arrestin2 recruitment and receptor internalisation than CCL21, both chemokines showed a similar level of Gαi protein activation. Altogether, these findings indicate that CEI is a powerful method to analyse receptor signalling and biased agonism.

## 1. Introduction

Chemokine receptors are members of the rhodopsin-like class A G protein-coupled receptors (GPCRs). They regulate immune cell activation and migration, and play a fundamental role in tissue development and organisation [1,2,3]. These functions are elicited by receptor interaction with a specific subset of chemotactic cytokines, namely chemokines. Chemokine-activated receptors, with the exception of atypical chemokine receptors (ACKRs), initiate downstream intracellular signalling mainly through activation of heterotrimeric Gαiβγ proteins that are sensitive to pertussis toxin (PTX) [2,3,4]. Receptor activation leads to G protein-coupled receptor kinase (GRK)-mediated phosphorylation of the receptor’s C-terminus, which enables the recruitment of β-arrestins. These multifunctional proteins initiate short-term receptor desensitisation and internalisation, and regulate downstream signalling processes. ACKRs (i.e., ACKR1-4) are devoid of functional G protein coupling but preserved the ability to recruit β-arrestins upon receptor stimulation [5].

Within the human chemokine signalling system, consisting of about 20 chemokine receptors and 50 chemokines, substantial promiscuity exists. Many chemokine receptors interact with more than one chemokine and often a given chemokine can stimulate multiple receptors. Although this was initially seen as signalling redundancy, it is now appreciated that, for several members of the chemokine receptor family, ligand bias occurs naturally, which contributes to the finetuning of chemokine signalling [6]. Biased agonists preferentially activate one or several pathways over others, for instance by primarily inducing G protein activation over β-arrestin recruitment. The phenomenon of biased agonism adds an additional layer of complexity to GPCR pharmacology studies but may provide new opportunities in drug discovery as it forms a conceptual basis for the development of agonists with higher therapeutic efficacy and/or a lower risk of side effects [7,8,9,10]. 

The human C-C chemokine receptor type 7 (CCR7) is a prime example of a chemokine receptor for which ligand bias was described. CCR7 is essential for homing various immune cells to secondary lymphoid organs and defining their position within the organ architecture [11,12,13,14]. CCR7 is stimulated by two natural ligands, C-C chemokine ligand 19 (CCL19) and CCL21, that only share a 32% sequence identity. Moreover, CCL21, unlike CCL19, has a positively charged 32 amino acid C-terminal tail that allows for avid binding to cell surface glycosaminoglycans (GAGs). Additionally, efficient CCL21 signalling requires CCR7 to be polysialylated, a posttranslational modification that releases CCL21 from a tail-regulated auto-inhibitory conformation [1,15,16,17]. CCR7 signalling bias has been extensively studied in vitro. While it seems to be the consensus that CCL19 is more potent in recruiting β-arrestins and inducing internalisation than CCL21, reports regarding G protein activation are ambiguous as it is still under debate whether they activate G proteins with similar potency and efficacy [17,18,19,20,21,22,23].

Studying chemokine receptor signalling is complex, especially when receptors are activated by multiple ligands. Determination of potential ligand bias generally requires the comparison of chemokines in multiple cellular assays, each looking into a separate signalling cascade or pathway. An alternative approach to determine ligand bias could be to employ assays with a readout that encapsulates the chemokine receptor response in its entirety, rather than comparing subsets of signalling pathways over multiple assays. Cellular electrical impedance (CEI) is a label-free, real-time cellular analysis methodology for which the readout reflects an integrated cellular response. CEI measures changes in the impedance of cells that adhere to a gold microelectrode surface. When these cells are subjected to a small alternating electrical current, they impede this current at the electrode–solution interface. The magnitude of the impedance depends on various cellular properties such as cell morphology, cell–cell contacts and cell adhesion. GPCR signalling induces morphological changes through G protein coupling-dependent rearrangement of the actin cytoskeleton [24]. As such, CEI-based assays are capable of capturing GPCR-specific responses in diverse cellular backgrounds as well as distinguishing between specific G protein couplings [24].

In this study, we investigated to what extent a CEI-based assay can capture the natural ligand bias previously described for human CCR7. For this, CCR7 was stably expressed in human embryonic kidney (HEK293) cells that were subsequently used for both CEI-based assays as well as more conventional label-based assays (i.e., β-arrestin recruitment, receptor internalisation, cAMP modulation and G protein activation). Our data show that CEI is capable of detecting specific CCR7-mediated signalling events with clearly distinct response profiles for the two endogenous ligands (CCL19 and CCL21). Detailed analysis and modelling of the differential CEI responses for CCL19 and CCL21 and comparison with data obtained from the more conventional label-based assays led us to conclude that CEI could be a valuable alternative methodology to identify and study ligand bias at GPCRs.

## 2. Results

**Figure 1:**
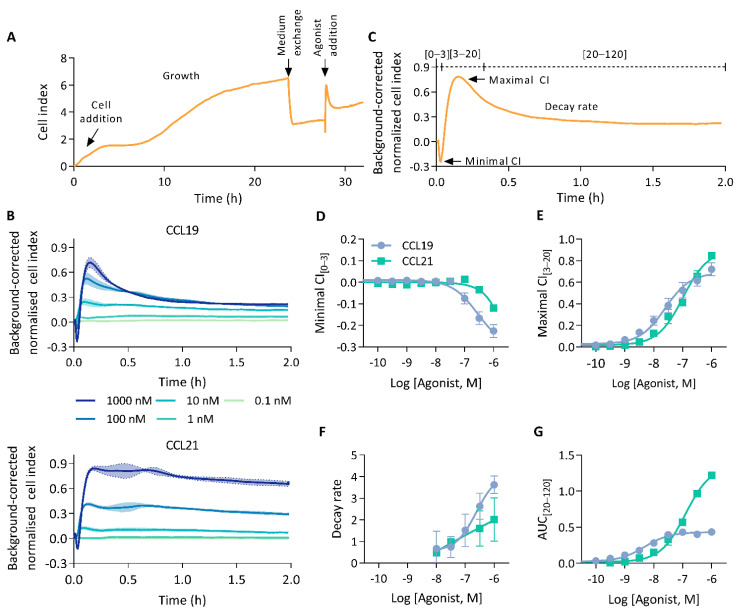
The CCR7 impedance profile. (**A**) Experimental overview of an impedance profile measurement. Cells were seeded and grown for 20 to 24 h. Thereafter, the growth medium was exchanged for serum-free medium and 4 h later cells were stimulated with agonist. (**B**) CEI measurements in HEK293 cells stably expressing CCR7 following stimulation with a CCL19 or CCL21 dilution series. Data are represented as the mean (line) and SD (shaded region) of three independent experiments with two technical replicates each. (**C**) Representation of relevant impedance profile features. The impedance profile is divided into three bins from which the minimal and maximal cell index (CI), and area under the curve (AUC) can be determined. (**D**–**G**) Dose–response curves of (**D**) minimal CI_[0–3]_, (**E**) the maximal CI_[3–20]_, (**F**) the decay rate and (**G**) the AUC of the final bin (AUC_[20–120]_). Data are represented as the mean and SD of three independent experiments with two technical replicates each. Curves were fitted to a three-parametric non-linear regression model except for (**F**) which was fit to a four-parametric model.

We investigated the capability of CEI to measure human CCR7 agonist-induced signalling. To this end, HEK293 cells stably expressing CCR7 were seeded in E-plates and an impedance profile was acquired following treatment with either CCL19 or CCL21 (Figure 1A). Stimulation with either chemokines resulted in an immediate, dose-dependent, and multi-facetted CEI response sharing broad pattern similarities but also clear differences (Figure 1B). Ligand addition resulted in a brief burst of transient negative cell index (CI) changes. Subsequently, after reaching a global minimum, the CI rose sharply to a maximum and decayed until it stabilised above baseline. At higher agonist concentrations, CCL21 displayed a “head-and-shoulder” profile reaching a second maximum shortly after the first, before initiation of the signal decay. This CCL21-induced shoulder appeared to be delayed and more pronounced with increasing ligand concentrations. 

HEK293 cells not expressing CCR7 did not respond to CCL19 addition, which validated that the observed response was indeed CCR7-specific. However, CCL21 did display a small increase in CI over time in these cells (Supplementary Figure S1).

To facilitate a quantifiable, in-depth analysis of the generated impedance profiles, we divided them into three bins, marking the negative transient phase [0–3 min], the rapid increase to the primary maximum [3–20 min], and the following signal decay [20–120 min] (Figure 1C). Thereafter, we sought to identify and assess various unique profile features. Although negative minimal CI_[0–3]_ values were observed for both CCL19 and CCL21, they were more pronounced upon CCL19 stimulation (Figure 1D). CCL19 and CCL21 responses reached their maximal CI_[3–20]_ within 10 min of ligand addition (Figure 1B). Where CCL19 was more potent, reaching the maximal CI_[3–20]_, CCL21 displayed slightly higher efficacy (Figure 1E; Table 1). This difference in efficacy most likely resulted from the lower minimal CI_[0–3]_ induced by CCL19. Indeed, adding maximal CI_[3–20]_ and the absolute value of the minimal CI_[0–3]_ resulted in identical efficacies for both agonists and brought the potencies closer together (Table 1). CCL19 displayed a rapid signal decay towards baseline levels. CCL21, on the other hand, showed no such kinetics, with its decay being more tempered (Figure 1B). To quantify this difference in signal decay we fitted a one-phase decay model starting from the global maximum. Decay rates increased proportionally to the ligand concentration for both chemokines. However, CCL19 exhibited a faster and stronger return to basal levels (Figure 1F). Signal decay could be approximated by the area under the curve (AUC) values calculated from the last bin (AUC_[20–120]_). CCL21 reached higher AUC_[20–120]_ values than CCL19, which rapidly reached a plateau (Figure 1G). As such, while maximal CI_[3–20]_ increased dose-dependently for CCL19, AUC_[20–120]_ values plateaued at lower agonist concentrations. This might be indicative of a stronger desensitisation process. In addition to ligand discrepancies, different aspects of the individual impedance profiles seem modulated by distinct molecular underpinnings. For instance, the potency difference between CCL19 and CCL21 becomes significantly larger when calculations are based on AUC_[0–120]_ compared to maximal CI_[3–20]_ (Table 1). Furthermore, maximal CI_[3–20]_ values increased proportionally with agonist concentrations (Figure 1E). In contrast, minimal CI_[0–3]_ values decrease proportionally (Figure 1D).

**Figure 2:**
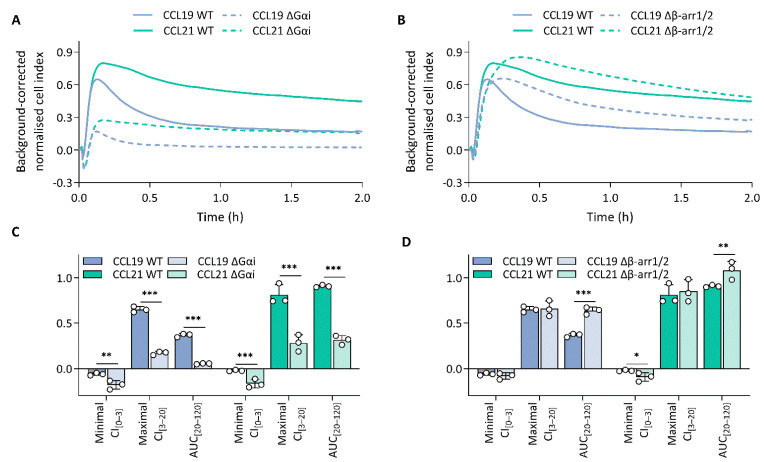
Gαi-mediated signalling dominates the impedance profile and β-arrestins modulate it. (**A**,**B**) CCR7 impedance profiles induced by CCL19 (100 nM) or CCL21 (250 nM) in CCR7-expressing wild-type (WT) and (**A**) Gαi knockout (KO) (ΔGαi) or (**B**) β-arrestin1/2-KO (Δβ-arr1/2) HEK293 cells. Data are represented as the mean (line) of three independent experiments with two technical replicates each. SDs are not shown for visual clarity. (**C**,**D**) The effect of (**C**) Gαi-KO (ΔGαi) and (**D**) β-arrestin1/2-KO (Δβ-arr1/2) on the minimal CI_[0–3]_, maximal CI_[3–20]_ and AUC_[20–120]_ following treatment with either CCL19 (100 nM) or CCL21 (250 nM). Data are represented as the mean (bar) and SD of three independent experiments (points) with two technical replicates. A one-way ANOVA followed by a Dunnett multiple comparison was used to assess the effect of the KOs compared to the wild-type cells. *, **, and *** represent *p* < 0.05, 0.01 and 0.001, respectively.

**Figure 3:**
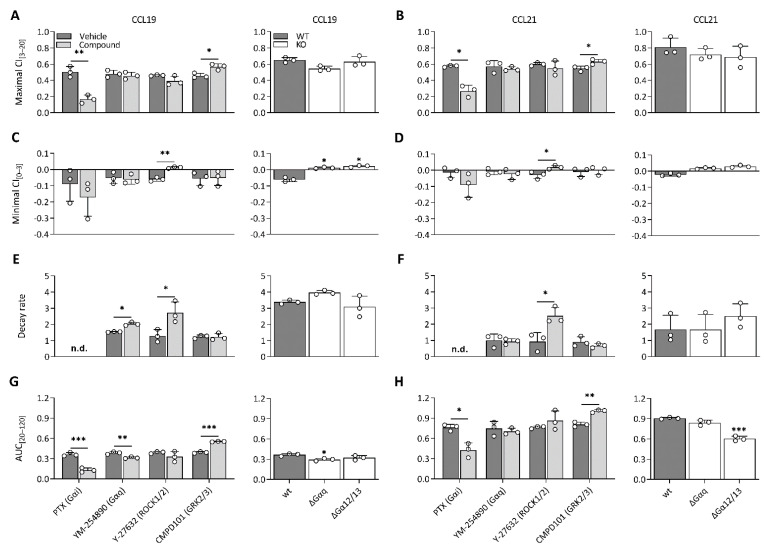
CCR7 impedance profiles are differentially modulated by underlying transducer couplings. (**A**–**H**) Effect of various compounds and KOs on (**A**,**B**) maximal CI_[3–20]_, (**C**,**D**) minimal CI_[0–3]_, (**E**,**F**) decay rate, (**G**,**H**) and AUC of the last bin (AUC_[20–120]_) following stimulation with CCL19 (100 nM) or CCL21 (250 nM) in CCR7-expressing HEK293 cells. (**A**–**H**, **left**) Cells were pre-treated with pertussis toxin (PTX) (50 ng/mL), YM-254890 (2 µM), Y-27632 (10 µM) or CMPD101 (10 µM) and stimulated with CCL19 or CCL21. The effect of a compound was analyzed against its specific vehicle control using an unpaired t-test with Welch’s correction. (**A**–**H**, **right**) Wild-type cells, Gαq-KO (ΔGαq) and Gα12/13-KO (ΔGα12/13) were stimulated with CCL19 or CCL21. A one-way ANOVA followed by a Dunnett multiple comparison was used to assess the effect of various KOs compared to the wild-type cells. (**A**–**H**) Data are represented as the mean (bar) and SD of three independent experiments (points) with two technical replicates. *, **, and *** represent *p* < 0.05, 0.01 and 0.001, respectively. Not determined = n.d.

To unravel which molecular processes comprise the CCL19 and CCL21 impedance profiles, we employed pharmacological modulation and knockout (KO) cell lines to map the effect of different transducers and their respective signalling pathway. To limit the effect of variable receptor expression levels, all KO variants were stably transfected with CCR7 and sorted for similar expression to the CCR7-expressing wild-type HEK293 cells (Supplementary Figure S2). Due to the nature of chemokine receptor signalling, we assumed that Gαi signalling would make up a considerable portion of the impedance profile. To assess this possibility, we performed experiments in Gαi-KO cells and pre-treated wild-type cells with PTX, which inhibits Gαi-coupling through adenosine diphosphate ribosylation and thereby successively inhibits adenylate cyclase activity as well. Genetic loss of Gαi (Figure 2A,C) and PTX pre-treatment (Figure 3A–H) resulted in the ablation of a significant portion of the CCL19 and CCL21 impedance profile. However, the lack of Gαi signalling did not entirely abrogate the response. Some positive changes remained while the minimal CI_[0–3]_ became more pronounced for both chemokines (Figure 2A,C and Figure 3A–D). Next, we investigated if the distinct agonist-mediated decay rates could be explained by the differential β-arrestin recruitment reported in the literature. While the loss of β-arrestin1/2 did not affect the maximal CI_[3–20]_ value itself, it did cause a minor temporal right shift, most likely due to a slower desensitisation onset (Figure 2B,D). Furthermore, CCL19-induced decay rates in β-arrestin1/2-KO cells and the associated AUC_[20–120]_ values were significantly smaller (two-way ANOVA, *p* < 0.001) and larger (Figure 2D), respectively. In contrast, decay rates mediated by CCL21 exposure were only slightly reduced (two-way ANOVA, *p* < 0.01); however, this still resulted in an AUC_[20–120]_ increase, although it was less significant than for CCL19 (Figure 2D). CMPD101, a specific GRK2 and GRK3 inhibitor, did not affect signal decay but did significantly increase the maximal CI_[3–20]_ and AUC_[20–120]_ (Figure 3A,B,E–H). As stated before, though Gαi signalling contributed significantly to the CCL19 and CCL21 impedance response, a residual non-Gαi dependent response remained. To investigate the contribution of other potential CCR7-Gα couplings to the CEI response, we utilised the Gαq inhibitor YM-254890 and opted to block ROCK1/2 signalling with Y-27632 as a downstream proxy of Gα12/13. Unlike Y-27632, which completely abolished any negative transient changes, YM-254890 had no impact (Figure 3C,D). We further corroborated these data by using a Gα12/13-KO cell line, which led to a solely positive impedance profile (i.e., lacking negative CI values) upon CCR7 stimulation (Figure 3C,D). Interestingly, loss of a functional Gαq protein, in contrast to its pharmacological inhibition, did induce similar effects to Y-27632 on the negative transient phase (Figure 3C,D).

**Figure 4:**
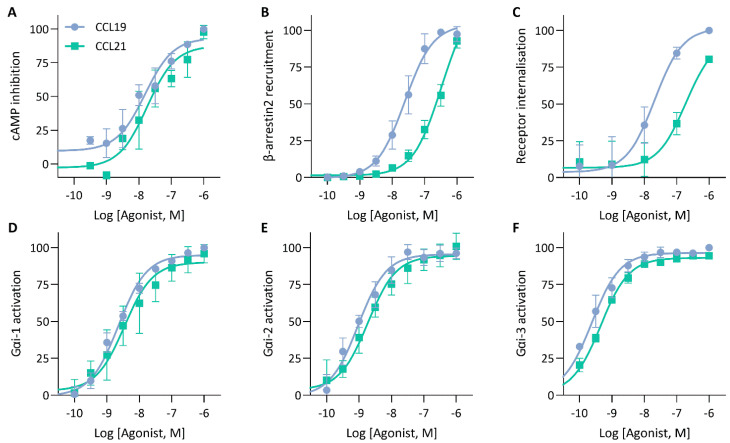
cAMP inhibition, G protein activation and β-arrestin recruitment by CCR7. (**A**) Analysis of ligand-mediated inhibition of forskolin-induced cyclic adenosine monophosphate (cAMP) production. CCR7-expressing HEK293 cells transiently transfected with a pGloSensor-22F were exposed to increasing concentrations of CCL19 or CCL21 and changes in bioluminescence were monitored (**B**) Measurement of β-arrestin2 recruitment to CCR7. HEK293 cells were transiently transfected with CCR7-LgBiT and β-arrestin2-SmBiT to monitor the recruitment of β-arrestin2 to CCR7 following CCL19 or CCL21 stimulation (**C**) Ligand-induced CCR7 internalisation. CCR7 was stained using a PE-labelled anti-CCR7 antibody and CCR7-fluorescent signal was monitored using flow cytometry and compared to an unstimulated control. (**D**,**E**) Assessment of G protein activation by CCR7. HEK293 cells stably expressing CCR7 were transiently co-transfected with (**D**) Gαi1-91-NLuc, (**E**) Gαi2-91-NLuc or (**F**) Gαi3-91-NLuc and Gγ2-LSS-mKATE2. G protein dissociation was monitored following stimulation with CCL19 or CCL21. (**A**–**F**) Data were scaled with the AUC (or median (**C**)) of the maximal CCL19 response set to 100 %. Data are represented as the mean and SD of three (or four (**B**)) independent experiments with each three (or one (**C**)) technical replicates. Curves were fitted to a three-parametric non-linear regression model.

**Figure 5:**
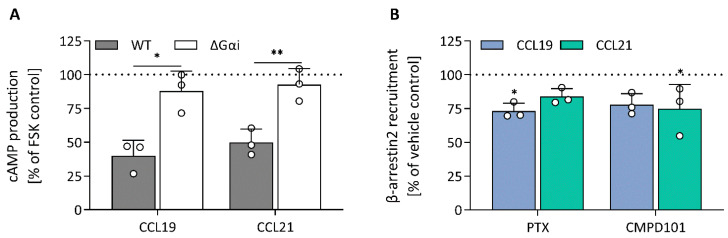
Modulation of cAMP production and β-arrestin2 recruitment. (**A**) The effect of Gαi-KO on CCL19- (63 nM) and CCL21- (79 nM) mediated cAMP production was investigated using stable CCR7-expressing HEK293 wild-type and Gαi-KO cells transiently transfected with pGloSensor-22F. The effect of the Gαi-KO (ΔGαi) was analysed against the wild-type cells using an unpaired t-test with Welch’s correction. (**B**) HEK293 cells were transiently transfected with CCR7-LgBiT and β-arrestin2-SmBiT and cells were treated with PTX (50 ng/mL) or CMPD101 (10 µM) prior to ligand (CCL19 (93 nM) or CCL21 (837 nM)) exposure. Changes in bioluminescence were monitored to assess compound effect on ligand-induced β-arrestin recruitment. A one-way ANOVA followed by a Dunnett multiple comparison was used to assess the effect of PTX and CMPD101 compared to the vehicle control set at 100%. (**A**,**B**) Data are represented as the mean and SD of three independent experiments with each two or three technical replicates. * and ** represent *p* < 0.05 and 0.01, respectively.

To validate the mechanisms that could explain the similarities and differences in the impedance profile induced by CCL19 and CCL21, we investigated CCR7-mediated signalling and function by employing various label-based assays. To minimize system bias, all these experiments were performed in the same cellular background used for the CEI experiments. First, as a proxy for Gαi activation, agonist-dependent inhibition of forskolin-induced cyclic adenosine monophosphate (cAMP) production was measured in HEK293 cells stably expressing CCR7 transiently transfected with a cAMP GloSensor plasmid. Both CCL19 and CCL21 induced robust Gαi activation with similar efficacy and potency (Figure 4A; Table 2). As expected, performing the assay with CCR7-expressing Gαi-KO cells completely abolished the agonist-induced inhibition (Figure 5A). Additionally, we assessed the direct activation of different Gαi-subunits using a nanoBRET-based biosensor approach. To this end, we fused Nluc to Gαi-1, Gαi-2 and Gαi-3 at position 91, in accordance with the recent literature [25,26,27], and transiently co-expressed each of them with Gγ2 N-terminally fused with LLS-mKate2 in stable CCR7-expressing HEK293 cells. In this system, CCL19 and CCL21 induced robust G protein activation of all tested Gαi isoforms with similar efficacy and potency (Figure 4D–F; Table 2).

To monitor CCR7-β-arrestin2 recruitment, HEK293 cells transiently co-transfected with a NanoBiT complementation system were used, in which CCR7 was C-terminally fused with LgBiT and β-arrestin2 was N-terminally fused with SmBiT. We found that CCL19 was significantly more potent than CCL21 with a 1.17-log potency difference (Figure 4B; Table 2). Although CCL21-mediated β-arrestin2 recruitment was not saturated at the highest concentration, a theoretical E_max_ value similar to CCL19 could be predicted. When cells were treated with the specific GRK2/3 blocker CMPD101, there was minimal β-arrestin2 recruitment inhibition for both chemokines (Figure 5B). Furthermore, PTX had no substantial effect on β-arrestin2 recruitment, suggesting that G protein activation is not essential for the recruitment of β-arrestin2 (Figure 5B). Lastly, since β-arrestins are pivotal in regulating GPCR internalisation, we questioned whether the observed differences in β-arrestin2 recruitment would perpetuate differential receptor internalisation. CCR7-expressing cells were exposed to varying concentrations of chemokines for 30 min at 37 °C, after which cell-surface receptor expression was quantified and compared to an unstimulated control. In line with the results obtained with the β-arrestin2 recruitment assay, both ligands induced a similar level of internalisation, but CCL19 was 13.8-fold more potent than CCL21 (Figure 4C; Table 2).

## 3. Discussion

The human CCR7 receptor was previously shown to be differentially activated by its natural ligands, CCL19 and CCL21. Although some conflicting data exist concerning the level of G protein activation induced by both chemokines, it is well established that CCL19-induced CCR7 activation results in more potent β-arrestin recruitment and receptor internalisation compared to CCL21 [19,20,21,22,23]. In this study, we analysed CCR7 signalling using CEI and demonstrate that this label-free technology holds promise as a methodology to investigate and decipher GPCR signalling. The CEI readout is not a priori focused on one particular signalling pathway but reflects a holistic cellular response combining the cellular effect of multiple individual signalling events. Importantly, the CEI response reflected the ligand bias for CCL19 and CCL21 indicating that CEI can detect differential receptor activation. The biological mechanisms behind this ligand-dependent discrepancy between the impedance profile of CCL19 and CCL21 could be explained by results obtained with more conventional label-based assays, which were also performed in HEK293 cells to minimize system bias across the different experimental readouts. Both CCL19 and CCL21 elicited a similar CEI response during the initial response phase, with the exception of a transient negative dip that was more pronounced when CCR7 was stimulated with high concentrations of CCL19. Ligand-induced impedance profiles following the negative impedance changes were dominated by Gαi signalling since they were largely abolished by PTX pre-treatment and genetic loss of Gαi. In line with this observation, the dose-dependent increase in maximal CI_[3–20]_ values for both ligands corresponds well with the results reported in our cAMP production and Gαi-biosensor assay. Early studies reported similar findings [19,20]. Two more recent studies, however, showed that there was a difference in potency, but not efficacy, between CCL19 and CCL21 concerning cAMP modulation [22,23]. In the latter studies, CCR7 signalling was studied in Chinese hamster ovary cells. It is important to note that the receptor interactome, which hinges on the relative abundance of intracellular interaction partners and modulators, might be different in these cells. Moreover, in contrast to our data, Corbisier et al. reported a difference in potency and efficacy between CCL19 and CCL21 in CCR7-expressing HEK293 cells using both a cAMP modulation assay and a direct G protein biosensor approach [21]. Currently, we cannot explain why their results differ from ours.

Agonist responses diverged significantly after reaching the maximal CI_[3–20]_. In contrast to CCL19, CCL21 displayed a head-and-shoulder profile, which was more pronounced at higher concentrations, and a slower signal decay was observed. The faster decay observed for CCL19 correlated well with the increased level of β-arrestin recruitment and CCR7 internalisation observed in the label-based assays. Moreover, when CCL19-induced impedance profiles were recorded in β-arrestin1/2-KO HEK293 cells the signal decay was significantly slower. As a result, in this cellular background, the CCL19 profile was more reminiscent of the CCL21 profile. In line with these findings, Watts et al. showed that CXCR3 ligands less effective in recruiting β-arrestin also displayed a prolonged CEI response as well as a more pronounced shoulder [28]. In contrast, when a synthetic β-arrestin superagonist (VUF10661) was tested, no shoulder was visible and the return to baseline occurred more quickly. In addition, stimulation of CXCR7, an ACKR known to recruit β-arrestins, but devoid of G protein activation, did not generate detectable CEI responses [29]. Altogether, it appears that β-arrestins alter G protein-mediated impedance profiles, but based on current data, cannot induce impedance changes independently of G protein activation. Hence, analysis of the impedance profiles can enable the quantification of differential G protein and β-arrestin activation by ligands acting on the same receptor. CCL21 strongly interacts with GAGs expressed on the cell surface via its C-terminal extension [30]. It is likely that this interaction is at the root of the slight time-dependent increase in CI seen when challenging wild-type CCR7-negative HEK293 cells with high CCL21 concentrations. This CCR7-independent GAG interaction may counteract a stronger signal decay that would be expected due to the increased β-arrestin recruitment and internalisation occurring at higher CCL21 concentrations. Furthermore, it was recently postulated that the CCL21–GAG interaction might result in the formation of a local reservoir from which CCL21 is released over time, coinciding with persistent but weaker CCL21-mediated signalling [17].

Promiscuous G protein couplings to GPCRs are not uncommon. We found that, although Gαi signalling was the dominant contributor to the CEI responses, Gαq and Gα12/13 also influenced the CEI profile, though to a lesser extent. ROCK kinases, downstream of Gα12/13, are involved in regulating cell morphology and CCR7-mediated migration [31]. Genetic loss of Gα12/13, as well as pharmacological ROCK1/2 inhibition by Y-27632 resulted in the abrogation of the negative transient phase. Furthermore, retraction of the trailing edge, which is attributed to Gα12/13-mediated RhoA activation, seems to require PLCβ-induced calcium mobilisation [32]. A recent study implied the need for a functional and activated Gαq protein to induce calcium release via the Gαi-Gβγ-PLCβ-mediated pathway [33]. Surprisingly, loss of Gαq, but not its pharmacological inhibition, resulted in an exclusively positive CEI response. It is possible that, despite inhibition, Gαq still retains its functionality in Gαi-mediated calcium release, in contrast to Gαq KO, where Gαq is completely absent.

The presented study describes CCR7 signalling using CEI and label-based assays. We showed that the CCL19 impedance profile is biased towards β-arrestin recruitment compared to CCL21. Similarly, in our label-based assays, CCL19 and CCL21 induced Gαi activation with equal potency and efficacy, but CCL19 was significantly more potent at recruiting β-arrestin2 and inducing internalisation. Furthermore, we demonstrated that CEI is a valuable addition to the GPCR research repertoire. Not only can it discriminate between differential receptor activation, but in concert with pharmacological modulation and KO, CEI provides an opportunity to study receptor signalling from a top-down perspective, allowing identification of individual components that contribute to the overall receptor signalling profile. Since CEI operates without labels, it is also applicable to investigate signalling in primary cells, which is much harder to realise using label-based assays. If throughput increases in the future, we believe that CEI can support initial screenings for biased ligands, specifically due to the integrated nature of the assay.

## 4. Materials and Methods

### 4.1. Cell Lines, Plasmids, Reagents and Transfections

The HEK293 A parental cell line and KO clones (Table 3) were kindly provided by Dr. A. Inoue (Tohoku University, Sendai, Japan) [34,35,36,37]. pNLF1-N vector (#N1351), pGlosensor-22F cAMP plasmid (#E2301), and pBiT1.1-C and pBiT2.1-N (#N2014) were purchased from Promega. CCR7 (#CCR0700000), ARRB2 (#ARRB200001), Gαi-1 (#GNAI100000), Gαi-2 (#GNAI200000), Gαi-3 (#GNAI300000) and Gγ2 (#GNG0200000) in a pcDNA3.1(+) vectors were purchased from cDNA Resource Centre. pEF1alpha-IRES Vector (#631970) was purchased from Takara and pBABE-puro-NLS-LSSmKate2 was a gift from Vladislav Verkhusha (Addgene plasmid #34586) [38]. pcDNA3.1(+) CCR7 was used to generate stable CCR7-expressing HEK293 cells and KO variants with similar receptor expression. Receptor surface expression was validated by flow cytometry using PE mouse anti-human CCR7 (Clone 150503, BD Pharmingen) and PE mouse IgG2a κ isotype control (Clone G155–178, BD Pharmingen, San Diego, CA, USA). Non-transfected HEK293 cells were cultured in Dulbecco’s Modified Eagle Medium, high glucose (DMEM; #41965, Thermo Fisher Scientific, Waltham, MA, USA) supplemented with 10% fetal bovine serum (FBS; #10270106, Thermo Fisher Scientific), referred to as growth medium. CCR7-expressing HEK293 cells were cultured in the same growth medium, further supplemented with 500 µg/mL Geneticin (#10131, Thermo Fisher Scientific). CCL19 (#300-29B) and CCL21 (#300-35A) were ordered from PeproTech. Y-27632 dihydrochloride (#1254) and pertussis toxin (PTX; #3097) were purchased from Tocris (Bristol, UK), YM-254890 (#257-00631) from FUIJIFILM Wako Chemicals (Neuss, Germany) and CMPD101 (#HY-103045) from Med Chem Express (Sollentuna, Sweden).

Cellular transfections were performed in suspension per the manufacturer’s protocol. Briefly, 1.5 × 10^5^ cells per mL were transfected with 0.5 µg plasmid DNA per mL using a 3:1 FuGENE^®^ HD Transfection Reagent (#E2311, Promega, Madison, MD, USA) to DNA ratio. The FuGENE^®^ HD Transfection Reagent/DNA mixture contained 20 ng/µL DNA and was incubated for 10 min at ambient temperature before adding it to the cell suspension. When a co-transfection of two plasmids was carried out, half the DNA concentration was used per plasmid.

The transfection setup for nanoBRET-based G protein activation assay differed slightly and is explained below.

### 4.2. Cellular Electrical Impedance Assay

The xCELLigence Real-Time Cell Analyzer (RTCA) DP instrument (Agilent, Santa Clara, CA, USA) was used to measure changes in cellular impedance following ligand stimulation. Briefly, RTCA E-plate VIEW 16 plates with embedded golden electrodes (#300600880, Agilent, Santa Clara, CA, USA) were coated with 10 µg/mL fibronectin (#F2006, Merck, Darmstadt, Germany) for 30 min and air-dried for one hour. A mandatory reference measurement was performed with 50 µL of growth medium per well to establish background CI values for each well. Thereafter, HEK293 cells were seeded at a density of 30,000 cells/well in a final volume of 100 µL. E-plates were placed at room temperature for 15 min and then transferred to the xCELLigence RTCA instrument, located in an incubator at 37 °C and 5% CO_2_. Cellular growth was monitored overnight every 20 min until a steady state was reached after 20–24 h. Following overnight incubation, E-plates were washed with 100 µL serum-free DMEM, 100 µL serum-free DMEM was added to each well and cell stabilisation was measured each minute for 4 h. Before ligand addition, a short normalisation measurement consisting of 5 total measurements, one every 5 s, was performed. Thereafter, 25 µL ligand at a 5× concentration was added and receptor stimulation was measured every 20 s for 4 h. When investigating the effect of compounds (CMPD101, YM-254890 and Y-27632) on receptor stimulation, cells were washed and 80 µL serum-free DMEM was added. Forty minutes before the ligand addition, 20 µL of 5× concentrated compound was added. For PTX, which was incubated overnight, 25 µL of 5× concentrated compound was added two hours after seeding the cells.

### 4.3. cAMP Modulation Assay

HEK293 cells stably expressing CCR7 were transfected in suspension with pGlosensor-22F. Transfected cells were seeded at a density of 1.5 × 10^4^ cells/well in white, clear flat-bottom 96-well plates (#CLS3610, Merck) coated with 100 µg/mL poly-D-lysin (#2780, Merck, Darmstadt, Germany) and incubated for 48 h at 37 °C and 5% CO_2_. Next, cells were washed with CO_2_-independent medium (#18045-054, Thermo Fisher Scientific, Waltham, MA, USA) supplemented with 10% FBS and incubated in 100 µL CO_2_-independent medium/10%FBS further supplemented with 300 µM 3-isobutyl-1-methylxanthine (#I7018, Merck, Darmstadt, Germany) and 2% GloSensor cAMP reagent (#E1291, Promega, Madison, MD, USA) for 2 h at 37 °C. The plate was transferred to the FLIPR Tetra (Molecular Devices, San Jose, CA, USA) and baseline luminescence was measured for 30 s every 5 s. Thereafter, 25 µL of 5× ligand was added automatically to the cell plate by the FLIPR Tetra and changes in bioluminescence were monitored in real time for 10 min every 5 s. Next, 25 µL Forskolin (#F6886, Merck, Darmstadt, Germany) was added to a final concentration of 5 µM and changes in bioluminescence were monitored in real time for 40 min every 5 s. When required 25 µL, 5× concentrated PTX was added 24 h post-transfection.

### 4.4. G Protein Activation Assay

G protein activation was monitored using a modified NanoBRET protein:protein interaction system (Promega, Madison, MD, USA). NanoLuc cDNA was inserted into Gαi-1, Gαi-2 and Gαi-3 at position 91 in a pcDNA3.1(+) vector. Additionally, Gγ2 was N-terminally fused to LSS-mKATE2 and cloned into a pcDNA3.1(+) vector. HEK293 cells stably expressing CCR7 were transiently co-transfected in suspension. Briefly, pcDNA3.1(+) Gαi1-91-Nluc, pcDNA3.1(+) Gαi2-91-Nluc or pcDNA3.1(+) Gαi3-91-Nluc and pcDNA3.1(+) Gγ2-LSSmKATE2(N) were transiently co-transfected in suspension at a 1:10 donor-acceptor ratio using a 3:1 FuGENE^®^ HD Transfection Reagent to DNA ratio with a final acceptor concentration of 1 µg plasmid DNA per mL. The FuGENE^®^ HD Transfection Reagent/DNA mixture contained 10 ng/µL DNA and was incubated for 10 min at ambient temperature before adding it to the cell suspension. Transfected cells were seeded at a density of 3.0 × 10^4^ cells/well in white, clear flat-bottom 96-well plates coated with 100 µg/mL poly-D-lysin and incubated for 48 h at 37 °C and 5% CO_2_. Next, cells were washed with an assay buffer (Hank’s Balanced Salt Solution (HBSS; #14065, Thermo Fisher Scientific, Waltham, MA, USA), 20 mM HEPES buffer (#15630-080, Thermo Fisher Scientific, Waltham, MA, USA), 0.5% FBS, pH 7.4) and incubated with 90 µL of a 1:100 Nano-Glo^®^ Vivazine™ working solution (#N2581, Promega, Madison, MD, USA) for 45 min at 37 °C and 5% CO_2_. The plate was transferred to the FLIPR Tetra and allowed to stabilise for 15 min. Baseline BRET was measured for 15 s every 2.5 s. Thereafter, 10 µL of 10× ligand was added automatically to the cell plate by the FLIPR Tetra and changes in BRET were monitored in real time for 25 min every 2.5 s. Measurements in the FLIPR tetra were carried out using a 440–480 nm donor emission filter and a custom 615 nm AT600lp acceptor emission filter (#296420, Chroma, Bellow Falls, VT, USA).

### 4.5. β-Arrestin Recruitment Assay

β-arrestin recruitment was monitored using the NanoBiT PPI system (Promega). CCR7 and β-arrestin2 were C-terminally fused with LgBiT and N-terminally fused with SmBiT, respectively, and cloned into a pEF1α-IRES vector. HEK293 cells were transiently co-transfected in suspension with pEF1α-IRES CCR7-LgBiT(C) and pEF1α-IRES SmBiT-ARRβ2(N), as previously described. Transfected cells were seeded at a density of 1.5 × 10^4^ cells/well in white, clear flat-bottom 96-well plates coated with 100 µg/mL poly-D-lysin and incubated for 48 h at 37 °C and 5% CO_2_. Next, cells were washed with an assay buffer (HBSS, 20 mM HEPES buffer (#15630-080, Thermo Fisher Scientific, Waltham, MA, USA), 0.5% FBS, pH 7.4) and incubated with 100 µL of a 1:100 Nano-Glo^®^ Live Cell Substrate (#N2012, Promega, Madison, MD, USA) working solution for 40 min at 37 °C and 5% CO_2_. The plate was transferred to the FLIPR Tetra and baseline luminescence was measured for 30 s every 5 s. Thereafter, 25 µL of 5× ligand was added automatically to the cell plate by the FLIPR Tetra and changes in bioluminescence were monitored in real time for 40 min every 5 s. When required, 20 µL of 5× compound was added to the working solution Nano-Glo Live Cell Substrate. In the case of PTX, 25 µL of 5× concentrated compound was added after 24 h of incubation.

### 4.6. Receptor Internalisation Assay

HEK293 cells stably expressing CCR7 were harvested with 0.25% trypsin, incubated for 2 h at ambient temperature, and washed twice with 2 mL DPBS (#14190, Thermo Fisher Scientific, Waltham, MA, USA) containing 2% FBS. Thereafter, cells were resuspended in 100 µL DPBS/2%FBS at 2 × 10^5^ cells per condition in a Falcon^®^ 5 mL round bottom polystyrene tube (#352054, Corning, NY, USA). Cells were stimulated with 25 µL 5× agonist for 30 min at 37 °C and 5% CO_2_. Next, cells were quickly washed twice with and resuspended in 2 mL and 100 µL ice-cold DPBS/2%FBS, respectively. Cells were stained with 5 µL PE mouse anti-human CCR7 (Clone 150503, BD Pharmingen), PE mouse IgG2a κ isotype control (Clone G155–178, BD Pharmingen) or DPBS/2%FBS and incubated for 1 h on ice. Cells were washed twice with 2 mL ice-cold DPBS and, finally, were resuspended in 200 µL ice-cold DPBS and kept on ice. Fluorescence was measured using the BD FACS Celesta (BD Bioscience) and data were processed with the BD FACSDiva Software (BD Bioscience) and FLOWJO™ version 10 (BD Bioscience).

### 4.7. Data Analysis

Raw relative light units (RLU) (cAMP modulation assay and β-arrestin recruitment assay), raw CI values (CEI assay) or a BRET ratio (G protein activation assay) were used as a starting point for data manipulations. BRET ratios were first calculated by dividing acceptor RLU by donor RLU values. All data were normalised to the baseline before ligand addition to reduce inter-well variation, an approach commonly used for kinetic fluorescent and bioluminescent readouts. This baseline was defined as the mean of a 5-point run-in time before ligand addition. Normalisation was performed by dividing all timepoints following ligand addition by the initial baseline. The technical replicates of these normalised readouts were averaged and then background-corrected by subtracting the values of their respective vehicle controls at each timepoint yielding a normalised background-corrected measurement. Dose-response curves were fitted to three parameters-log(agonist) vs. response model in GraphPad V9.3.1 (GraphPad Software, San Diego, CA, USA) unless otherwise stated. Decay rates were fitted from the global maximum with a one-phase decay model in R version 4.0.5 using the SSasymp function from the *stats* (version 3.6.2) R package. Statistical analysis was performed as described in figure or table legends.

## Figures and Tables

**Table 1:** Overview of the potency and efficacy of CCR7 ligands in label-free cellular electrical impedance assay. Data are representative of the mean and SD of three independent experiments with two technical replicates. The difference between EC50 and Emax was analysed using an unpaired t-test with Welch’s correction. *, **, and *** represent *p* < 0.05, 0.01 and 0.001, respectively. ns indicates no significant difference was detected.

Assay	Ligand	Potency pEC50 (M) ± SD		Efficacy Emax ± SD	
Maximal CI_[3–20]_	CCL19	7.59 ± 0.12	**	0.69 ± 0.05	*
	CCL21	6.99 ± 0.12		0.90 ± 0.01	
Maximal CI_[3–20]_+ |Minimal CI_[0–3]_|	CCL19	7.29 ± 0.11	**	0.94 ± 0.07	ns
	CCL21	6.84 ± 0.12		1.07 ± 0.04	
AUC_[0–120]_	CCL19	8.20 ± 0.07	***	0.58 ± 0.02	***
	CCL21	6.93 ± 0.09		1.57 ± 0.05	

**Table 2:** Overview of the potency and efficacy of CCR7 ligands in label-based assays. Data are representative of the mean and SD of three independent experiments. The difference between EC50 and Emax was analysed using an unpaired t-test with Welch’s correction. **, and *** represent *p* < 0.01 and 0.001, respectively. ns indicates no significant difference was detected.

Assay	Ligand	Potency pEC50 (M) ± SD		Efficacy (% of CCL19) Emax ± SD	
cAMP production	CCL19	7.83 ± 0.39	ns	94.34 ± 3.77	ns
	CCL21	7.67 ± 0.42		91.48 ± 11.92	
β-arrestin recruitment	CCL19	7.61 ± 0.20	***	104.68 ± 3.74	ns
	CCL21	6.48 ± 0.15		122.30 ± 12.50	
Internalisation	CCL19	7.70 ± 0.15	**	101.81 ± 1.99	**
	CCL21	6.73 ± 0.07		94.26 ± 1.65	
G protein activation—Gαi-1	CCL19	8.64 ± 0.06	ns	95.03 ± 1.02	ns
	CCL21	8.44 ± 0.37		90.98 ± 6.94	
G protein activation—Gαi-2	CCL19	9.02 ± 0.15	ns	95.69 ± 3.51	ns
	CCL21	8.72 ± 0.21		94.78 ± 8.16	
G protein activation—Gαi-3	CCL19	9.61 ± 0.16	ns	96.66 ± 1.70	ns
	CCL21	9.34 ± 0.05		93.12 ± 1.64	

**Table 3:** Cell line overview and their respective gene KO.

Cell Line	Knocked-Out Gene
HEK293	None
HEK293-ΔGαi	GNAI1, GNAI2, GNAI3, GNAO1, GNAZ, GNAT1, GNAT2
HEK293-ΔGαq	GNAQ, GNA11
HEK293-ΔGα12/13	GNA12, GNA13
HEK293-ΔARRβ1/2	ARRB1, ARRB2

## Data Availability

Data are contained within the article or Supplementary Materials.

## Supplementary Materials

The supporting information can be downloaded at: https://www.mdpi.com/article/10.3390/ijms23168903/s1.
