# Supplementary material for: Cellular Electrical Impedance as a Method to Decipher CCR7 Signalling and Biased Agonism
Source: Int J Mol Sci. 2022 Aug 10;23(16):8903. doi: 10.3390/ijms23168903 (PMC9408853; doi:10.3390/ijms23168903)
Supplement: Supplementary file 1 [file ijms-23-08903-s001.zip › ijms-1841246-supplementary.pdf]

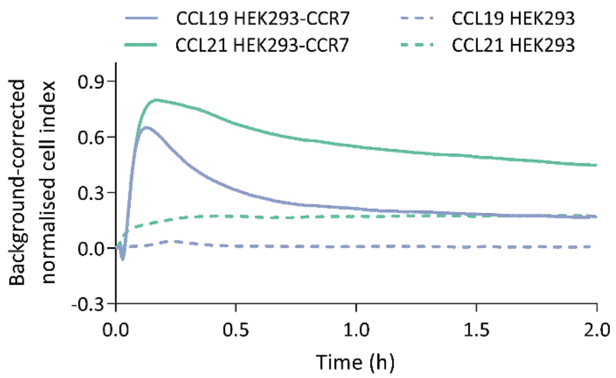

Supplementary Figure S1. CCL21 induces a small CEI response in HEK293 cells without CCR7. *CCR7 impedance profiles induced by CCL19 (100 nM) or CCL21 (250 nM) in HEK293 cells and HEK293 cells stably expressing CCR7. Data are represented as the mean (line) of three independent experiments with two technical replicates each. SDs are not shown for visual clarity.*

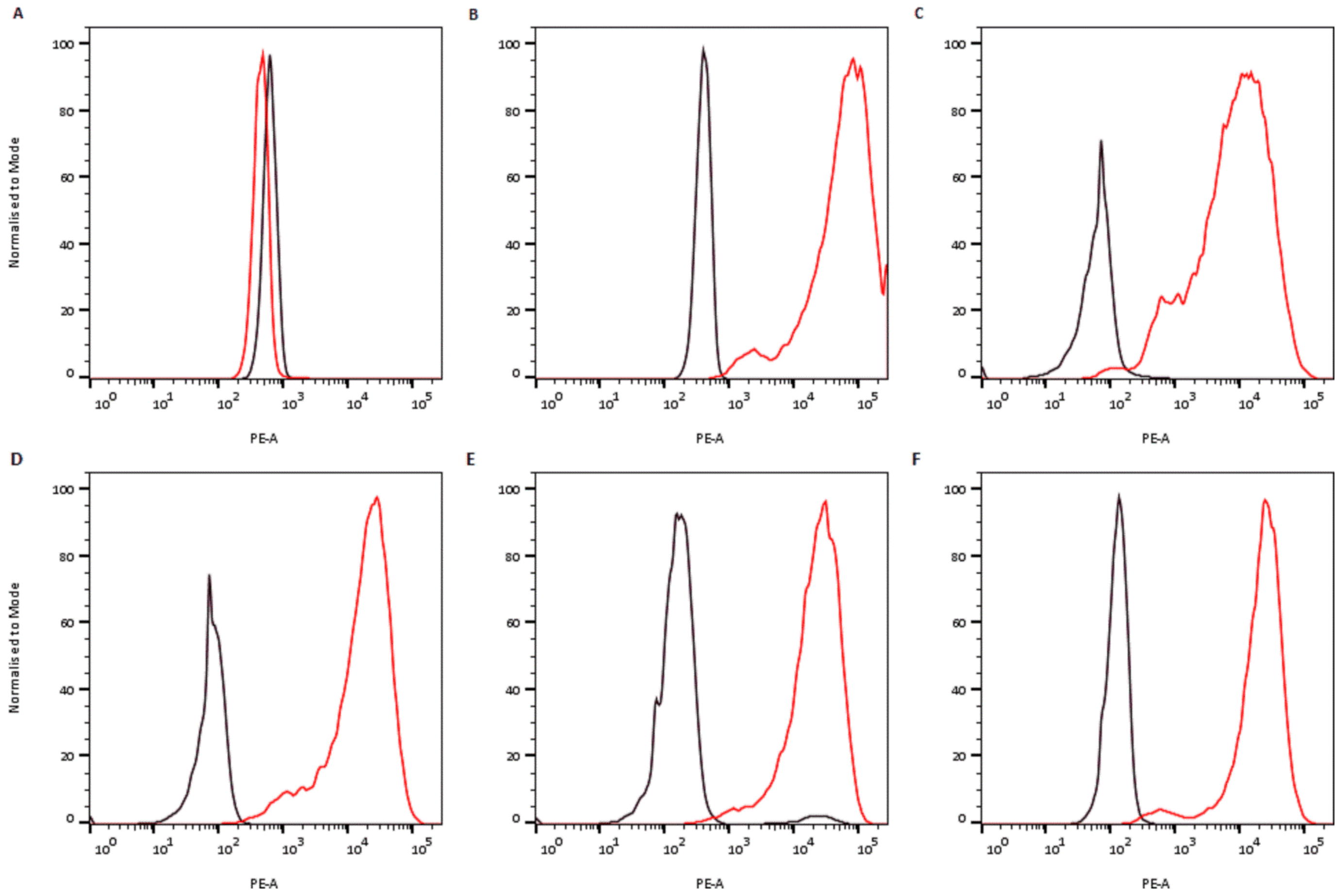

***Supplementary Figure S2. Expression of CCR7 on employed cell lines**. **(A)** HEK293, **(B)** HEK293 CCR7, **(C)** HEK293 ΔGαi CCR7, **(D)** HEK293 ΔGαq CCR7, **(E)** HEK293 ΔGα12/13 CCR7, **(F)** HEK293 Δβ-arr1/2 CCR7 were stained with PE Mouse anti-Human CCR7 (red) or PE Mouse IgG2a κ Isotype Control (black) and receptor expression was quantified using flow cytometry.*
